# Current update on toll-like receptors and prostate cancer: a decade of progress and emerging insights

**DOI:** 10.3389/fimmu.2025.1711815

**Published:** 2026-01-12

**Authors:** Jahnavi Janjirala, Matthew Blanton, Kenneth S. Ramos, Dekai Zhang

**Affiliations:** 1 Center for Inflammatory and Infectious Diseases, Institute of Biosciences and Technology, Texas A&M University, Houston, TX, United States; 2 Center for Genomic and Precision Medicine, Institute of Biosciences and Technology, Texas A&M University, Houston, TX, United States

**Keywords:** cancer immunity, immune checkpoint inhibitor, immunotherapy, innate immunity, prostate cancer, toll-like receptors

## Abstract

Prostate cancer remains the most common cancer and the second leading cause of cancer-related mortality among men in the United States. Despite advances in therapy, relapses and progression of disease continue to pose major clinical challenges, underscoring the need for innovative strategies. A decade ago, we reviewed the emerging evidence linking Toll-like receptors (TLRs), a family of innate immune sensors, to prostate cancer initiation and progression. Since then, substantial advances have deepened our understanding of TLR biology in prostate cancer, revealing their role in either promoting tumor growth or activating anti-tumor immunity depending on cellular context and signaling pathways. Recent studies have expanded knowledge of TLR expression on both immune and tumor cells, identified key endogenous ligands driving chronic inflammation, and uncovered microRNA-mediated regulation of TLR signaling. Moreover, new insights into TLR polymorphisms and their potential association with cancer risk, as well as preclinical and clinical progress in TLR-targeted immunotherapies, highlight both opportunities and challenges in translating TLR biology into therapeutic applications. In this review, we update the field by summarizing the latest discoveries, evaluating the complexities of TLR signaling in prostate cancer, and discussing how this evolving knowledge may inform future biomarker development and immunotherapeutic strategies.

## Introduction

Cancer remains a major global health challenge. According to a recent report, the United States alone will see approximately 2,041,910 new cancer diagnoses and 618,120 cancer-related deaths in 2025 (1). The disease arises from uncontrolled cell proliferation within the body. Multiple factors contribute to cancer development across different tissue types, including exposure to physical and chemical carcinogens, as well as bacterial and viral infections (1–4). These microbial agents play a critical role in the pathogenesis of several malignancies, including lung, colorectal, hepatic, cervical, and prostate cancers (5–9).

According to the most recent cancer statistics, prostate cancer is projected to be the most frequently diagnosed cancer and the second leading cause of cancer-related death among men in the United States in 2025 (1). Current treatments such as chemotherapy and radiotherapy prolong survival; however, relapses and metastasis are frequent and often fatal outcomes. Moreover, conventional therapies lack selectivity, targeting both malignant and healthy cells and thereby causing significant toxicity. These limitations underscore the urgent need for more precise and less harmful therapeutic interventions.

Immunotherapy has emerged as a promising approach, offering potential to both prevent tumor progression and improve treatment outcomes. Because evasion of immune surveillance is a hallmark of cancer, prostate tumors employ multiple mechanisms to escape immune detection. In this context, immune dysfunction and chronic inflammation play central roles in carcinogenesis (10). Chronic inflammation, often linked to microbial infections, is increasingly recognized as a major contributor to prostate cancer initiation and progression (11). Indeed, a growing body of evidence highlights the impact of inflammation on disease progression and the importance of targeting immune pathways as a therapeutic strategy (12).

Among immune modulators, Toll-like receptors (TLRs), a family of pattern recognition receptors central to innate immunity, have attracted particular interest. By orchestrating inflammation and antimicrobial responses, TLRs directly influence the tumor microenvironment and prostate cancer development. Approximately a decade ago, we reviewed the emerging evidence linking TLRs to prostate cancer (13). In this updated review, we summarize recent advances, highlight the evolving understanding of TLRs in prostate cancer, and discuss potential directions for future research and therapeutic development.

## Toll-like receptor: the first identified family of pattern recognition receptors in innate immunity

The discovery of Toll-like receptors (TLRs) evolved through several pivotal findings. In 1989, Charles Janeway proposed that pattern recognition receptors (PRRs) must recognize pathogen-associated molecular patterns (PAMPs) to activate innate immunity and trigger downstream cell signaling pathways (14). This theoretical framework gained experimental support in 1996 when Hoffman Lab demonstrated that the Toll gene from *Drosophila* was essential for antimicrobial response (15, 16). In the following year, Janeway’s Lab identified mammalian homologs to the Toll gene and demonstrated that the first mammalian Toll-like receptor, TLR4, could activate NF-κB signaling (16). In 1998, Beutler Lab identified TLR4 as the receptor responsible for recognizing bacterial LPS (15, 16). Collectively, these discoveries established the fundamental role of TLRs in pathogen recognition and innate immune activation.

Toll-like receptors are a class of transmembrane proteins that serve as essential components of the innate immune system. They function by detecting pathogen-associated molecular patterns (PAMPs), which are conserved structures present in bacteria, viruses, fungi, and parasites (12, 17). In addition to microbial recognition, TLRs can also sense damage-associated molecular patterns (DAMPs), which are released during various pathological conditions, including cancer (12, 17).

**Figure 1:**
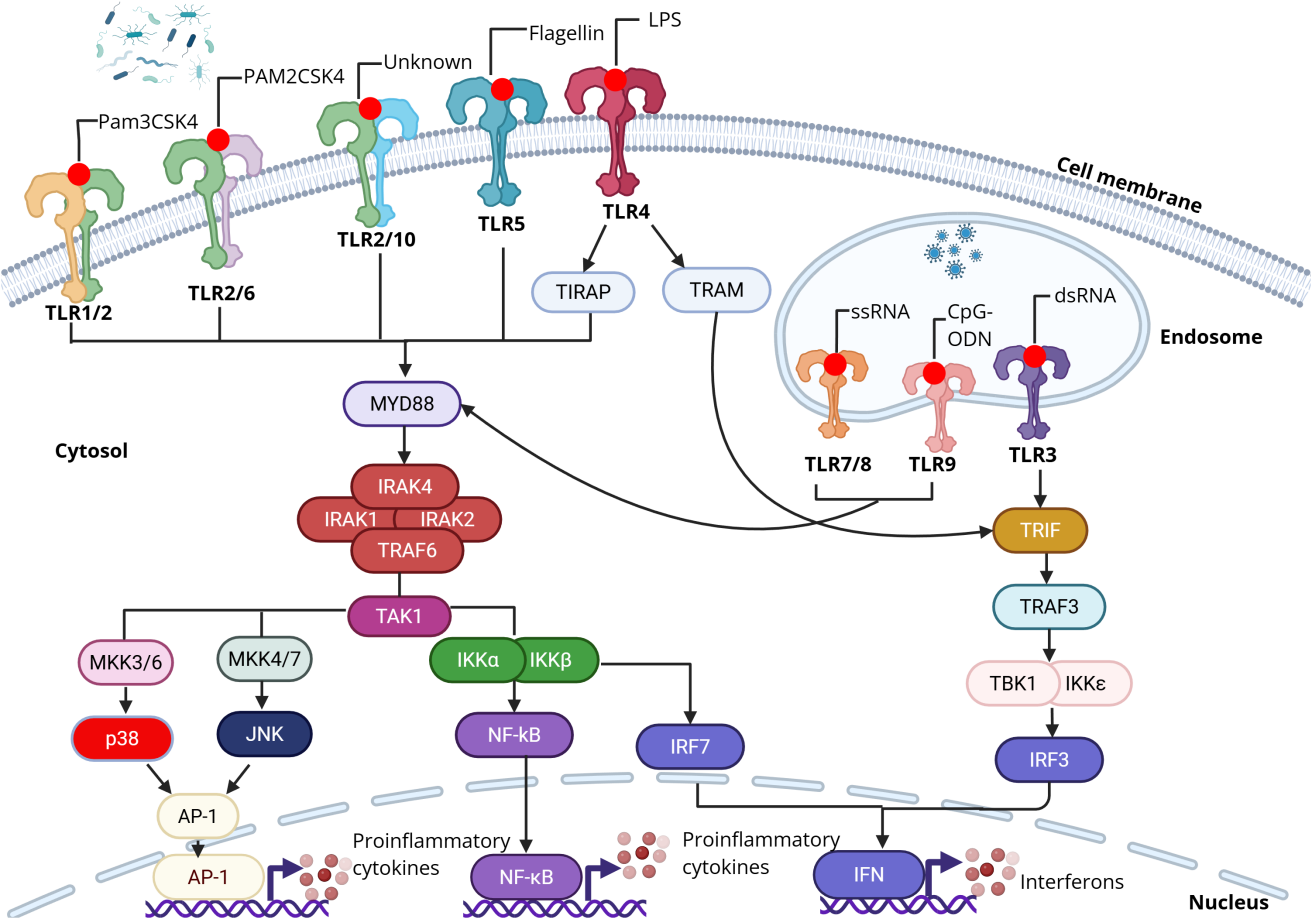
TLR activation and TLR-mediated signaling. TLRs recognize pathogen associated-molecular patterns (PAMPs). Cell surface TLRs (e.g., TLR1/2, TLR2/6, TLR2/10, TLR5, and TLR4) generally recognize PAMPs from bacteria such as lipopeptides, flagellin, and LPS. Endosmoal TLRs (TLR3, TLR7/8, and TLR9) generally recognize nucleotides from viruses such as double-stranded RNA, single-stranded RNA, and CpG DNA. Upon activation, TLRs activate downstream NF-κB through the MyD88 pathway which leads to NF-κB translocation to the nucleus and upregulates production of pro-inflammatory cytokines. TLR3 or TLR4 can activate the TRIF pathway resulting in induction of type 1 interferons. Created with BioRender.

To date, ten TLRs have been identified in humans. Among them, TLR1, TLR2, TLR4, TLR5, and TLR6 are primarily located on the cell surface, whereas TLR3, TLR7, TLR8, and TLR9 are confined to endosomal compartments (**Figure 1**). TLR10 is primarily on the cell surface although several studies have reported endosomal localization (17, 18). TLR2 might heterodimerize with either TLR1 or TLR6. Also, TLR10 might be heterodimerized with TLR2 (19). Each TLR recognizes a different ligand with a high degree of specificity. Bacterial ligands are recognized by cell surface TLRs, whereas viral ligands, mainly nucleic acids, are recognized by endosomal TLRs. The heterodimerization of TLR2 with TLR1 or TLR6 recognizes bacterial lipoproteins. TLR3 is activated by double-stranded RNA or its synthetic analog polyinosinic–polycytidylic acid [poly(I:C)], while TLR4 responds to lipopolysaccharides (LPS). TLR5 detects flagellin, TLR7/TLR8 detects single-stranded RNA, and TLR9 detects CpG-rich DNA motifs (CpG-ODN). TLR10 remains an orphan receptor with an undefined function and signaling pathway, although a recent study indicated a potential ligand of TLR10 (20).

Once activated, TLRs initiate downstream signaling by engaging one or more of four adaptor proteins: myeloid differentiation factor 88 (MyD88), TICAM1 (also known as TRIF), TIRAP (also known as MAL), and TICAM2 (also known as TRAM and TIRP). These signaling pathways are divided into MyD88-dependent and TRIF-dependent. With the exception of TLR3, all TLRs, and the IL-1 receptor family, signal predominantly through the MyD88 adaptor. In contrast, TLR3 exclusively utilizes the TRIF-dependent pathway, while TLR4 is capable of activating both MyD88- and TRIF-mediated signaling (12). Engagement of TLRs ultimately activates multiple downstream cascades, including NF-κB, MAPKs, JNKs, p38, ERKs, and interferon regulatory factors (IRF3, IRF5, and IRF7), producing pro-inflammatory cytokines (21). Activation of TLRs in antigen-presenting cells (APC) also triggers adaptive immunity by promoting the activation and differentiation of T cells (22). TLRs have been implicated in the regulation of cell death by promoting the expression of several anti-apoptotic proteins, including Bcl-2–related protein A1 (BCL2A1), cellular inhibitor of apoptosis proteins 1 and 2 (cIAP1 and cIAP2), X-linked inhibitor of apoptosis (XIAP), and additional members of the Bcl-2 family (23).

## TLR expression and function in prostate cancer

**Figure 2:**
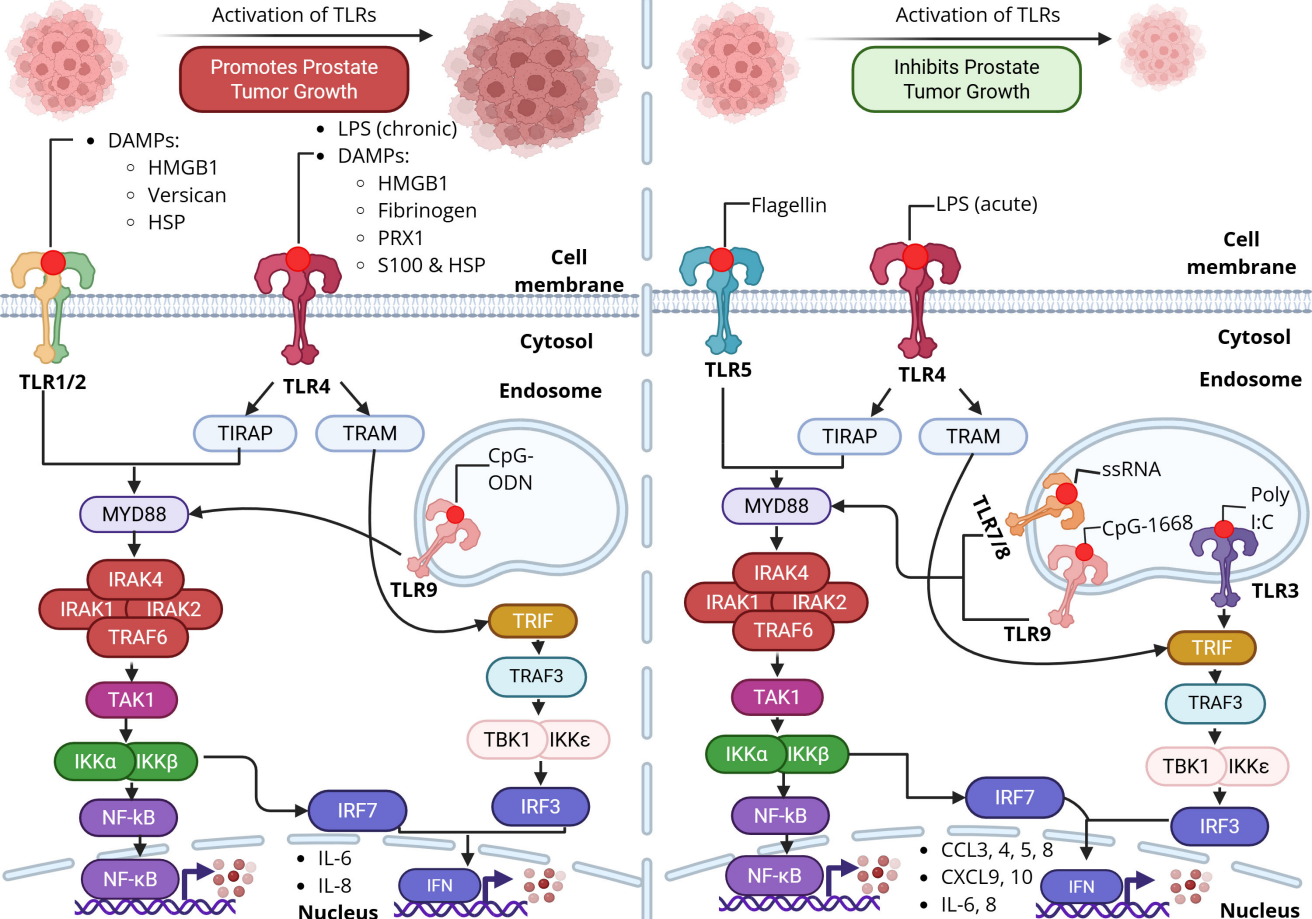
Toll-like receptor signaling in prostate cancer. The role of TLR activation in prostate cancer is complex, being either tumor promoting or inhibiting. Activation of TLRs1/2 and TLR4 by DAMPs enhances NF-κB-mediated transcription of pro-inflammatory cytokines. For example, activation of TLRs 4, 5, 9, and 3 can induce anti-tumor responses. These TLRs act through either the MyD88 or TRIF pathway to stimulate NF-κB and IRF transcription factors, driving production of type 1 interferons and chemokines to inhibit prostate tumor growth. Created with BioRender.

Toll-like receptors are primarily expressed on innate immune cells, including dendritic cells, macrophages, and natural killer (NK) cells. Their activation in these cells triggers innate immune responses, driving the production of pro-inflammatory cytokines, chemokines, and adhesion molecules, which subsequently promote the initiation of adaptive immunity (24). Interestingly, increasing evidence indicates that TLRs are also expressed on tumor cells. TLR signaling within tumor cells, as well as within the tumor microenvironment through innate immune cells, creates a complex regulatory network (**Figure 2**). Consequently, TLR activation can exert a “double-edged sword” effect, both promoting and inhibiting carcinogenesis (**Table 1**).

**Table 1:** The role of toll-like receptors (TLRs) and their activated signaling in prostate cancer.

TLRs	Ligands	The role in prostate cancer	Reference
TLR1/2	HMGB1, Versican HSP	Promotes tumor growth	(27, 28, 32, 33, 35)
TLR3	Poly I:C	Inhibits tumor growth	(23, 24, 35, 48)
TLR5	Flagellin	Inhibits tumor growth	(27, 35, 39)
TLR7/8	ssRNA	Inhibits tumor growth	(35)
TLR4	HMGB1, LPS Fibrinogen, PRX1 S100 proteins HSP	DAMPs and chronic LPS promote tumor growth and acute LPS inhibits tumor growth	(29–35, 42, 43, 58)
TLR9	CpG-1668 CpG-ODN	CpG-ODN promotes tumor growth and CpG-1668 inhibits tumor growth	(22, 35, 47, 59, 60)

Recent studies reveal that prostate cancer harbors a distinct microbiome enriched in Gram-negative Proteobacteria, suggesting a tumor microenvironment populated with bacterial ligands capable of activating TLR4 (25). Notably, Ma et al. identified several Gram-positive taxa, including *Pediococcus pentosaceus*, *Listeria monocytogenes*, *Lactobacillus crispatus* ST1, and *Bacillus halodruans*, that were inversely correlated with Gleason score (26). While direct evidence in prostate cancer remains limited, these Gram-positive bacteria are typically recognized by TLR2 heterodimers, which detect lipoteichoic acids and lipoproteins, and are frequently associated with anticancer functions (26). Conversely, the Gram-negative species *Nevskia ramosa* showed a positive correlation with Gleason score, suggesting it likely promotes tumor progression through TLR4 activation through LPS (26). Several taxa demonstrated negative associations with TNM stage, including *Rhodococcus erythropolis PR4*, *D. acidovorans SPH-1*, *Methylobacterium radiotolerans JCM 2831*, *Stenotrophomonas maltophilia K279a*, and *Meiothermus silvanus DSM 9946* (26). With the exception of *Rhodococcus* these bacteria are predominately gram-negative and therefore likely engage both TLR4 and TLR2 signaling pathways.

In prostate cancer, identifying a specific pathogen responsible for initiating TLR signaling is often challenging. Instead, endogenous TLR ligands, particularly damage-associated molecular patterns (DAMPs) released from necrotic or stressed tissues, are thought to play a central role. Among these, high-mobility group box 1 (HMGB1) has been shown to activate TLR2 and TLR4 (27), while the extracellular matrix proteoglycan versican functions as a TLR2 agonist (28). Additionally, peroxiredoxin 1 (Prx1) has been implicated as a TLR4 agonist in prostate cancer progression (29). S100 proteins (e.g., S100A4, S100A8, and S100A9) activate TLR4 (30, 31). Heat shock proteins (HSPs), particularly HSP70 and HSP90, also serve as DAMPs and can activate TLR2 and TLR4 (32, 33). Recent studies have also shown that fibrinogen (another TLR4 ligand) is associated with poor prognosis of prostate cancer (34).

Activation of certain TLRs may exert inhibitory effects on prostate cancer growth (**Figure 2**). In prostate cancer, activation of TLR2, TLR4, and TLR9 stimulates tumor growth while activation of TLR3, TLR4, TLR5, and TLR7 suppress tumor development (35). This highlights that TLR activation in prostate cancer is a “double-edged sword” effect. Notably, TLR3 has been detected in prostate cancer cells (22, 24, 36). Expression of TLR3 mRNA has been reported in the LNCaP, PC3, and DU145 prostate cancer cell lines, with stimulation by poly(I:C) leading to a marked increase in TLR3 transcript levels, indicating a potential functional role in tumor biology (24). At the protein level, TLR3 was observed in both LNCaP and DU145 cells at comparable levels, whereas expression was somewhat lower in PC3 cells. TLR3 is upregulated in prostate cancer (19). Treatment with poly(I:C) rapidly induced NF-κB–dependent expression of inflammatory mediators. Conditioned media from poly(I:C)-treated LNCaP and DU145 cells recruited leukocyte subpopulations, suggesting that TLR3 activation may modulate early immune responses within the tumor microenvironment (13). *In vivo*, poly(I:C) stimulation markedly inhibited prostate tumor growth, likely through enhanced infiltration of T lymphocytes and NK cells in a type I interferon–dependent manner (24). Analysis of human prostate cancer samples revealed that 85 of 112 tumors exhibited TLR3 expression, and elevated TLR3 levels were significantly correlated with an increased risk of cancer recurrence (37). In contrast, there is another report that claims that lower TLR3 expressions have been associated with prostate cancer recurrence in prostate cancer tissues compared to benign prostatic hyperplasia (BPH) tissues (19). TLR3 activation induces metabolic reprogramming in PCa cells by upregulating glycolytic genes, increasing HIF-1α–dependent lactate production, and exacerbating the Warburg effect (21). Moreover, a recent study showed that prostate cancer cells secrete the Transient Receptor Potential Cation Channel Subfamily M Member 8 (TRPM8) into extracellular vesicles, which then function as a TLR3 agonist (38). In prostate cancer cells, activation of the TLR3-NF-κB/IRF signaling cascade by TRPM8 triggers a sterile inflammatory response (38). This TRPM8-mediated inflammation promotes NK cell infiltration, thereby enhancing antitumor immunity activity in prostate cancer (38). TLR5 is expressed in LNCaP and DU145 cells, and its activation induces the production of chemokines that recruit immune effector cells, including NK cells and cytotoxic CD8^+^ T cells, which are likely to contribute to tumor suppression (39). It has been proven by Liang and colleagues that PCa patients with high expression of TLR5 had a better prognosis than those with low expression of TLR5 (27).

Activation of certain other TLRs may have distinct effects on prostate cancer progression (**Figure 2**). TLR4 expression has been documented in multiple animal models, including constitutive expression in epithelial cells of the rat ventral prostate, a rat adenocarcinoma cell line, and primary prostate cultures (40, 41). In humans, TLR4 is present in DU145 and PC3 cell lines, as well as in normal prostate tissue in stroma and epithelium (42, 43). TLR4 expression is lower in LNCaP cells compared to PC3 cells (35). Furthermore, TLR4 has been detected in clinical prostate cancer specimens. TLR4 expression is upregulated in prostate cancer (19). Immunohistochemical analysis revealed a significant correlation between TLR4 expression and proliferation/invasion capabilities of PCa cells, suggesting its potential role as a poor prognostic indicator (28). TLR9 was initially believed to be expressed exclusively in immune cells; however, recent evidence indicates that functional TLR9 is also present in various tumor types, including prostate cancer (37, 44). A clinical study has confirmed TLR9 expression in prostate cancer specimens (37). Joanna et al. reported TLR9 expression in human prostate cancer cell lines, including LNCaP, C4-2B, DU145, and PC3, as well as in clinical tumor samples, while it was absent in MDA Pca2b cells and stromal cells from adenocarcinoma specimens (45, 46). Notably, TLR9 levels were significantly higher in both the epithelial and stromal compartments of prostate cancer compared with benign prostatic hyperplasia, with the most pronounced expression observed in poorly differentiated tumors (22, 44). It is reported that TLR9 is upregulated in prostate cancer (19). TLR9 can be both tumor promoting and tumor suppressive in prostate cancer, and TLR9 is a marker for poor prognosis (35). It has also been reported that TLR9 expression in prostate cancer, promotes immune evasion through LIF mediated polymorphonuclear MDS activation and amplification (47). Therefore, targeting the TLR9/LIF/STAT3 signaling pathway with oligonucleotide-based inhibitors might offer new immunotherapies to treat prostate cancer (47). Sikora et al. found elevated TLR2 and TLR9 levels in more advanced stages of PCa (48). Fan and colleagues reported on the expression of TLR2 and TLR10 in prostate epithelial and stromal cells, with expression levels positively correlating with severity of inflammation. This study was the first to demonstrate TLR10’s inhibitory role in RWPE-1 cells, showing that knockdown of TLR10 significantly increased HMGB1, IL-6, and IL-8 secretion (49).

The roles and biological significance of TLRs in prostate cancer appear to be highly complex (**Figure 2**). It is possible that distinct and yet-uncharacterized TLR signaling pathways are engaged in tumor cells or innate immune cells during cancer progression, or that initial TLR activation in these cells influences subsequent signaling and effector responses. Further investigation into these mechanisms will be critical for evaluating the therapeutic potential of TLR agonists or antagonists as anti-cancer agents.

## MicroRNAs regulate TLRs in prostate cancer

MicroRNAs (miRNAs) are small non-coding RNA molecules, approximately 22 nucleotides in length, that regulate gene expression post-transcriptionally (41, 50). They bind to complementary sequences within the 3′ untranslated region (3′ UTR) of target mRNAs, resulting in gene silencing either through translational repression or mRNA degradation. Dysregulation of miRNAs has been implicated in both the initiation and progression of human cancers (19, 35, 47, 50–52). Specific miRNAs have been identified as key contributors to tumorigenesis, functioning either as oncogenes or as tumor suppressors (41, 53).

MicroRNAs are increasingly recognized as key regulators of TLR signaling (50–55). Recent studies have highlighted interactions between miRNAs and TLRs in the context of prostate cancer. For example, miR-29a functions as a potential tumor suppressor by regulating TRAF4 expression in metastatic prostate cancer (54). Furthermore, TLR3 activation by poly(I:C) induces miR-29b, miR-29c, miR-148b, and miR-152, which inhibit DNA methyltransferases and restore the expression of the tumor suppressor RARβ (53). A study revealed that a miR-21/TLR8 axis plays a role in prostate carcinogenesis (55). Additionally, miR-155 deficiency in a mouse model of autoimmune prostatitis mitigated chronic prostatic inflammation by inhibiting TLR4/NF-κB signaling, leading to decreased proinflammatory cytokines and oxidative stress (41). TLR activation can promote either inhibition or progression of prostate cancer. MicroRNAs are likely key regulators of TLR expression and signaling, thereby influencing prostate cancer development.

## TLR signaling in prostate cancer

The Toll-like receptor signaling pathway is well characterized in innate immune cells. Upon ligand binding, TLRs recruit one or more adaptor proteins, including MyD88, TRIF, MAL, and TRAM, via TIR domain interactions. With the exception of TLR3, most TLRs signal through the MyD88-dependent pathway. Engagement of MyD88 activates IL-1 receptor–associated kinase (IRAK), which interacts with tumor necrosis factor receptor–associated factor 6 (TRAF6), ultimately triggering MAPK and NF-κB signaling cascades. In contrast, TLR3 and TLR4 can also signal through a MyD88-independent pathway, in which TRIF recruitment leads to activation of NF-κB and type I interferon responses (46).

TLR3 can be activated in prostate cancer cells, where it has been shown to induce apoptosis and inhibit proliferation. In human LNCaP cells, TLR3 signaling partially mediates these effects via inactivation of the PI3K/Akt pathway, with involvement of Cyclin D1, c-Myc, p53, and NOXA (48). Additional studies indicate that HIF-1α promotes apoptosis through a PKC-dependent mechanism in poly(I:C)-treated prostate cancer cells. Furthermore, TLR3 activation by poly(I:C) stimulates JNK and p38 via PKC-α, leading to caspase-8-dependent apoptosis (22, 56). A recent study has further shown that poly(I:C) stimulation induces apoptosis via caspase-3/7 activation in a TLR3 expression-dependent manner, with chemoresistant DU145 and PC3 cells displaying heightened sensitivity (23). Gambara et al. reported that poly(I:C) induces IRF3 phosphorylation, leading to upregulation of Noxa (a BH3-only pro-apoptotic protein) and promotion of apoptosis; intriguingly, suppression of Noxa expression paradoxically augmented cell death (24). Poly(I:C) treatment in LNCaP cells upregulates chemokines (CCL3, CCL4, CCL5, CCL8, CXCL9, CXCL10) and NF-κB–dependent cytokines (IL-6, IL-12), promoting NK and CD8^+^ T cell recruitment (24). In the TRAMP model, this type I interferon-dependent immune infiltration contributes to effective tumor growth suppression (36). Conversely, Gambara’s study demonstrated that poly(I:C) treatment significantly suppresses the growth of LNCaP cells *in vivo* by inducing direct, non-immune-mediated apoptosis in prostate cancer cells (24). Although TLR3 is primarily associated with tumor suppression, its overexpression in PCa cells has been linked to increased migration and invasion via EGFR and ERBB2 signaling (23). The TLR5 agonist flagellin activates NF-κB signaling in LNCaP and DU145 cells, producing pro-inflammatory mediators.

TLR4 has the potential to both inhibit and promote tumor growth. TLR4 activation in prostate cancer cells promotes tumor progression: LPS stimulation in DU145 cells triggers NF-κB–dependent production of IL-6 and IL-1β, while in PC3 cells it enhances VEGF and TGF-β1 expression (42, 43). Conversely, siRNA-mediated knockdown of TLR4 in PC3 cells reduces cell migration and invasion (57). TLR4 signaling promotes ERG phosphorylation and transcriptional activation through the MAPK pathway (29). TLR4 and COX-2 expression are elevated in prostate cancer, and silencing their expression through p65 phosphorylation inhibition leads to tumor growth and invasion (58). TLR9 activation by CpG-ODN contributes to prostate cancer cell invasion, primarily through NF-κB activation and upregulation of COX-2 (59). Additionally, TLR9 expression has been shown to enhance invasiveness via induction of MMP-13 *in vitro* (60). In both studies, CpG-ODN stimulation did not alter cellular proliferation, indicating that TLR9 signaling primarily promotes cancer progression and metastatic potential. Moreira et al. identified NF-κB/RELA and STAT3 as two downstream effectors in TLR9 signaling that regulate the transcription of stem cell-related and tumor-propagating genes, such as *NKx3.1*, *BMI-1*, and *KLF4* (56). Although TLR9 is mostly associated with tumor promotion, recent findings show that treatment with synthetic agonist CpG-1668 suppresses prostate tumor growth by enhancing systemic innate immunity, marked by increased macrophages, CD8^+^ T cells, and neutrophils, and inducing macrophage-derived IFN-β that inhibits cancer cell proliferation (22).

While the signaling mechanisms of TLRs are well characterized, they do not completely account for the contrasting effects observed in prostate cancer, where activation of certain receptors such as TLR3 suppresses tumor growth, whereas others like TLR2 enhance it (**Figure 2**). This suggests that distinct downstream pathways may underlie these divergent outcomes.

## TLR gene polymorphisms and prostate cancer risk

Polymorphisms in TLR genes have been associated with susceptibility to a wide range of infectious and inflammatory diseases. TLR gene polymorphisms may influence prostate cancer risk by modulating inflammation, yet studies examining this association have produced inconsistent results (61, 62).

Several studies have reported associations between single nucleotide polymorphisms (SNPs) in TLR4 and prostate cancer risk (63–68). Variants within the TLR gene cluster TLR6-TLR1-TLR10 have also been implicated (69, 70). However, these findings are inconsistent. For example, Shui et al. found no significant correlation between 10 TLR4 SNPs and prostate cancer risk (71), and Chen et al. reported no association between TLR6-TLR1-TLR10 variants and prostate cancer susceptibility (72). Similarly, a meta-analysis by Lindström et al. failed to demonstrate a clear relationship between TLR gene polymorphisms and prostate cancer risk (73). Weng et al. published a meta-analysis in 2014 examining 20 TLR4 SNPs and found no significant associations with aggressive prostate cancer risk, consistent with previous meta-analyses on overall PCa (74).

A study by Lindström et al., reported that individuals with the T allele of the TLR4 polymorphism, Thr399Ile, are less likely to develop prostate cancer compared to individuals carrying the C allele (73). Also, individuals with the CC genotype of rs11536889 have a higher risk of developing prostate cancer compared to individuals with the GG genotype (73). A meta-analysis by Wang et al. reported that the TLR3 polymorphism, rs3775290 was found not to be associated with prostate cancer in an Asian cohort (75). Another study done by Dubey et al., reported that the TLR6_rs2381289 GA genotype in Jamaican black men was associated with an increased risk for developing prostate cancer, in both the unadjusted and age-adjusted linear regression models (76). Also, another study, by Duy et al., identified a TLR4 SNP in intron 3, rs52149356 T>G is associated with an increased risk of both PCa and benign prostatic hyperplasia (39). The study also found that carriers with the TLR4 SNP C.331–206 A>G had a statistically reduced risk for developing PCa (39).

Discrepancies in TLR polymorphism studies may reflect differences in detection methods, population ethnicity, and sample size. Larger, multi-ethnic studies are needed to clarify their diagnostic, prognostic, and therapeutic potential in prostate cancer.

## Targeting TLRs for prostate cancer immunotherapy

The capacity of TLRs to influence prostate cancer progression has spurred interest in developing TLR-targeted immunotherapies. Currently, three TLR-targeting drugs have received FDA approval for cancer treatment: Bacillus Calmette–Guérin (BCG), monophosphoryl lipid A (MPL), and imiquimod (77). BCG, an attenuated strain of *Mycobacterium bovis*, activates TLR2 and TLR4 and is used both as a tuberculosis vaccine and for treatment of *in situ* bladder carcinoma. MPL, derived from LPS and a potent TLR4 agonist, serves as an active component of Cervarix, targeting oncogenic human papillomavirus (HPV) (78, 79). Imiquimod, a synthetic imidazoquinoline acting via TLR7, is widely employed in the treatment of skin cancers such as basal cell carcinoma and Bowen’s disease (80–82). It stimulates the production of pro-inflammatory cytokines, including IFN-α, IL-6, and TNF-α, and activation of TLR7/8 promotes a Th1-mediated anti-tumor response dependent on IFN-γ (83, 84). Supporting this approach in prostate cancer, Han et al. demonstrated that imiquimod inhibits both human and murine prostate tumor growth by inducing apoptosis (85, 86).

Several preclinical and clinical studies are currently exploring the potential of TLR-based immunotherapies for prostate cancer. Activation of TLR3 directly induces apoptosis in human prostate cancer cells, highlighting TLR3 agonists as promising anti-tumor agents (56). For instance, Ampligen, a formulation of poly(I:C) acting as a TLR3 agonist, has demonstrated tumor growth inhibition in early clinical trials (87, 88). Hiltonol, or poly-ICLC, a particular formulation of poly(I:C), suggests a promising immunostimulatory mechanism when combined with dendritic cell vaccination and radiotherapy in advanced cancer patients (42), including metastatic castration-resistant prostate cancer (mCRPC) (43). A phase 2 clinical trial (NCT00514072) is currently evaluating the use of BCG vaccination for prostate cancer treatment. Additionally, the multi-peptide, dual-adjuvant telomerase vaccine GX301, which incorporates imiquimod, has demonstrated strong immunogenicity with minimal toxicity in PCa patients (89). A more recent phase II trial demonstrated the vaccine’s safety in mCRPC patients and showed improved immunological responses with a higher number of administrations (44). Additionally, TLR4 activation by LPS has been shown to promote chemoresistance to docetaxel in prostate cancer cells (90).

Recent evidence suggested that combining cabazitaxel, a chemotherapy agent for mCRPC, with poly(I:C) activates TLR3 signaling and suppresses prostate cell proliferation by inducing apoptosis (36). Another study showed that cabazitaxel activates TLR4 signaling as well, leading to increased NF-κB activity and apoptotic cell death *in vitro* (45). Mobilan is a recombinant adenoviral vector engineered for PCa immunotherapy that co-expresses human TLR5 and a secreted flagellin-based TLR5 agonist, stimulating innate immunity to suppress tumor growth (91). It has been evaluated in a human study and appears to be well tolerated (51). As TLR7/8 agonists, imidazoquinolines possess antitumor activity. BAIT628 exhibited efficacy in the TRAMP-C2 prostate cancer model (52), and resiquimod (R848) was effective in combination therapy (50).

A recent study found that treatment with nobiletin (NOB) on PC3 and LNCaP prostate cancer cell lines had immunotherapeutic capabilities. Treatment with increasing concentrations of NOB greatly decreased cell proliferation (35). The inhibitory effects induced by NOB treatment were more prevalent in LNCaP cells compared to PC3 (35). Treatment with NOB also lowered TLR4 and TLR9 expression in both cell lines (35). NOB treatment alone was found to suppress TLR4 and TLR9 expressions than when treated with NOB and LPS or CpG-ODN (35). While examining the correlation of NOB treatment with the TLR4/TRIF/IRF3 signaling pathway, NOB treatment alone lowered TLR4, TRIF, and IRF3 protein expression in PC3 cells (35). In LNCaP cells, NOB treatment alone only decreased TRIF and IRF3 protein levels (35). While examining the correlation of NOB treatment with the TLR9/IRF7 signaling pathway, TLR9 and IRF7 protein expression were inhibited following treatment with NOB alone in both PC3 and LNCaP cells (35). All together this shows that NOB treatment alone has a greater anti-inflammatory effect on the inhibition of both the TLR4/TRIF/IRF3 and TLR9/IRF7 signaling pathways in prostate cancer than when combined with LPS or CpG-ODN treatment.

## Limitations of the study

This review summarizes current knowledge on the role of TLRs and their signaling pathways in prostate cancer, but several limitations warrant consideration. First, while the prostate tumor microenvironment contains multiple families of pattern recognition receptors, our analysis focuses exclusively on TLRs, potentially overlooking important interactions with other innate immune receptors. Second, TLR signaling exhibits considerable complexity and context-dependency, demonstrating both tumor-suppressive and tumor promoting functions depending on the specific circumstances. Given the extensive body of literature, our review may not have captured all mechanistic insights comprehensively. Third, the rapidly expanding field of TLR-targeted immunotherapy means that some relevant studies, particularly recent therapeutic investigations, may have been inadvertently excluded. Future research integrating a broad spectrum of innate immune pathways alongside more comprehensive therapeutic analyses will be essential to fully elucidate the multifaceted role of TLRs in prostate cancer.

## Concluding remarks and future directions

Toll-like receptors are expressed in both immune cells and prostate cancer cells, where accumulating evidence demonstrates that their activation can either promote or suppress tumorigenesis depending on the signaling context. While studies examining TLR polymorphisms suggest potential associations with prostate cancer risk, the findings remain inconsistent and insufficient to establish definitive conclusions. Nonetheless, TLRs represent attractive therapeutic targets due to their capacity to enhance immune responses with minimal toxicity, particularly when used as adjuvants in combination regimens. Several TLR-targeted immunotherapies are currently being evaluated in prostate cancer, including cabazitaxel, mobilan, and resiquimod. Advancing our understanding of TLRs in prostate cancer will require elucidating how individual TLR pathways interact within the complex prostate tumor microenvironment, distinguishing beneficial from detrimental TLR activation patterns, and identifying robust biomarkers to guide patient-specific TLR-based therapeutic strategies.
